# Structural Defects on Graphene Generated by Deposition of CoO: Effect of Electronic Coupling of Graphene

**DOI:** 10.3390/ma17133293

**Published:** 2024-07-03

**Authors:** Cayetano Hernández-Gómez, Pilar Prieto, Carlos Morales, Aida Serrano, Jan Ingo Flege, Javier Méndez, Julia García-Pérez, Daniel Granados, Leonardo Soriano

**Affiliations:** 1 Departamento de Física Aplicada, Universidad Autónoma de Madrid, 28049 Madrid, Spain; cayetano.hernandez@uam.es (C.H.-G.);; 2 Instituto Nicolás Cabrera (INC), Universidad Autónoma de Madrid, 28049 Madrid, Spain; 3 Applied Physics and Semiconductor Spectroscopy, Brandenburg University of Technology Cottbus–Senftenberg, 03046 Cottbus, Germany; carlos.moralessanchez@b-tu.de (C.M.); flege@b-tu.de (J.I.F.); 4 Departamento de Electrocerámica, Instituto de Cerámica y Vidrio (ICV), CSIC, 28049 Madrid, Spain; aida.serrano@icv.csic.es; 5 Instituto de Ciencia de Materiales de Madrid, ICMM-CSIC, Sor Juana Inés de la Cruz 3, 28049 Madrid, Spain; jmendez@icmm.csic.es; 6 IMDEA Nanociencia, Faraday 9, 28049 Madrid, Spain; julia.garcia@imdea.org (J.G.-P.);

**Keywords:** graphene, CoO, structural defects, X-ray photoelectronic spectroscopy, confocal Raman microscopy

## Abstract

Understanding the interactions in hybrid systems based on graphene and functional oxides is crucial to the applicability of graphene in real devices. Here, we present a study of the structural defects occurring on graphene during the early stages of the growth of CoO, tailored by the electronic coupling between graphene and the substrate in which it is supported: as received pristine graphene on polycrystalline copper (coupled), cleaned in ultra-high vacuum conditions to remove oxygen contamination, and graphene transferred to SiO_2_/Si substrates (decoupled). The CoO growth was performed at room temperature by thermal evaporation of metallic Co under a molecular oxygen atmosphere, and the early stages of the growth were investigated. On the decoupled G/SiO_2_/Si samples, with an initial low crystalline quality of graphene, the formation of a CoO wetting layer is observed, identifying the Stranski-Krastanov growth mode. In contrast, on coupled G/Cu samples, the Volmer-Weber growth mechanism is observed. In both sets of samples, the oxidation of graphene is low during the early stages of growth, increasing for the larger coverages. Furthermore, structural defects are developed in the graphene lattice on both substrates during the growth of CoO, which is significantly higher on decoupled G/SiO_2_/Si samples mainly for higher CoO coverages. When approaching the full coverage on both substrates, the CoO islands coalesce to form a continuous CoO layer with strip-like structures with diameters ranging between 70 and 150 nm.

## 1. Introduction

The pioneering work of A. Geim and K. Novoselov in 2004 opened the door to a new fundamental and applied physics based on 2D materials, already proposing the possibility of using graphene’s outstanding behavior in real devices [1]. Formed by a 2D lattice of hexagon cells of sp^2^ hybridized carbon atoms, the resulting electronic structure provides graphene unique physical properties, such as high mechanical tensions (120–130 GPa) [2,3], superior thermal conductivity (5 kWm^−1^K^−1^) [4] and electrical conductivity (3000 times higher than Cu) [5], ballistic transport over the micrometer [6], and 2.3% visible light absorption [7]. Despite the extended use of chemical vapor deposition (CVD) in the synthesis of defect-free micrometric flakes of graphene [8], the scaling-up of graphene applications, unfortunately, has not yet been possible due to the well-known difficulty of obtaining low defective layers, especially when transferred to semiconducting and insulating substrates required by the semiconductor industry or when embedded into complex heterostructures.

Graphene heterostructures combined with functional oxides have received increasing attention in the last decade, as they served as an excellent platform to exploit the material properties of graphene. Several proposals for hybrid devices based on graphene and metallic oxides (TiO_2_, Fe_2_O_3_, and ZnO, among others) have already been made, including transistors [9,10], energy storage [11,12], solar cells [13,14], gas sensors [15], and catalysts [16]. In this context, cobalt oxides, commonly CoO (Co^2+^) and Co_3_O_4_ (Co^2+^ and Co^3+^) presenting rock salt and spinel crystalline structures, respectively [17], are very suitable candidates. Their high earth crust abundance, low environmental impact, and excellent reactivity, widely applied as catalysts [18,19], gas sensors [20], and in energy storage devices such as batteries and supercapacitors [21,22,23], make them quite attractive. Cobalt oxides exhibit antiferromagnetic properties that can be applied in multiple novel technologies, such as spintronic devices [24]. Moreover, their combination with graphene improves not only the graphene properties but also those of cobalt oxide. For example, cobalt oxide/graphene composites have already been used in Li-ion batteries as anode materials and in supercapacitors as electrodes, showing high cycle performance with low-capacity loss [25,26].

A deep understanding of the interaction between graphene and cobalt oxides remains hidden. In this context, several approaches using photoelectron-based spectroscopies have been followed by our group in previous works. Various studies have been conducted on the interface region and electronic interaction between cobalt oxides and substrates, where CoO ultrathin layers and nanoclusters were grown on highly oriented pyrolytic graphite (HOPG) and metal oxides such as SiO_2_, Al_2_O_3_, and MgO with different degrees of ionic/covalent bonds [27,28,29]. In particular, the growth of CoO on HOPG stands out as a first-step case of study due to its chemical and structural similarities with a graphene surface. In this case, CoO growth shows a Stranski-Krastanov growth mechanism, forming a well-ordered wetting layer on HOPG before the growth of CoO islands on top. In addition, X-ray photoelectron spectroscopy (XPS) and X-ray absorption near-edge structure (XANES) experiments shed light on the initial weakening of the graphite sp^2^ σ-bonds due to the strong interaction between cobalt and graphite top-layers [30], which results in the latter oxidation and development of sp^3^-like defects. The disturbance of the graphite bonds facilitates a more efficient carbon gasification reaction at lower temperatures than directly using metallic Co nanoclusters [31,32]. Similarly, the formation of a CoO wetting layer and the development of defects on the graphene net could be expected when depositing the metal oxide under the same conditions. However, the employed substrate to support graphene is also expected to play a critical role. It will modify graphene’s electronic properties, i.e., determining if it is electronically coupled to or uncoupled from the substrate and thus tuning its interaction with cobalt oxide deposits. Therefore, when studying CoO_x_ growth on graphene, it is also interesting to investigate the growth on various graphene/substrate systems to differentiate the role of graphene electronic properties as well as each of the components, i.e., substrate, graphene, and substrate/graphene and deposit/graphene interactions.

This work presents a study of the cobalt oxide/graphene interaction, focusing on how the initial electronic state of graphene, i.e., the electronic interaction of graphene with the substrate, affects the early stages of growth of CoO. For that, two types of graphene-based substrates are used: (i) pristine graphene on polycrystalline Cu sheets (G/Cu), where graphene has been directly grown by CVD and is electronically coupled to the metallic substrate, and (ii) graphene transferred onto silicon oxide substrates (i.e., thermally oxidized Si wafers, G/SiO_2_/Si), where, on the contrary, the graphene is electronically uncoupled to the SiO_2_/Si. The deposits have been in situ and stepwise characterized by XPS, giving insights into the growth model and changes in the oxidation state of all constituents related to the evolution, as a function of thickness and growth morphology, of the electronic interaction between substrate, graphene, and cobalt oxide. Further ex situ morphological and structural studies employing atomic force microscopy (AFM) and micro-Raman spectroscopy complement this analysis.

## 2. Materials and Methods

Graphene samples were grown by CVD on 25 µm thick polycrystalline Cu foil, with a 10 × 10 mm surface, labeled as G/Cu along the text. Graphene grown in the same conditions was transferred onto 290 nm thick thermally oxidized SiO_2_ substrates (SiO_2_/Si) via the polymethyl methacrylate route (PMMA) [33], referred to in the text as G/SiO_2_/Si samples. This process leads to electronically uncoupled graphene. In order to reduce the amount of Cu oxidation and intercalation of adventitious molecules, i.e., O_2_, H_2_O, etc., after atmosphere exposure during the transfer from the CVD reactor to the XPS cluster, the pristine G/Cu samples were annealed at 300 °C under ultra-high vacuum (UHV) conditions (3 × 10^−9^ mbar) for 30 min.

Subsequently, cobalt oxide (CoO) was grown on G/Cu and G/SiO_2_/Si samples by reactive thermal evaporation of metallic cobalt (99.999% purity) at room temperature (RT) and in an oxygen (O_2_) atmosphere at 3 × 10^−5^ mbar. The e-beam evaporator (Tectra, Frankfurt, Germany) used was placed 10 cm from the samples and perpendicular to their surface, with negligible contribution of the evaporator cell to the substrate temperature. Depending on the substrate, a deposition rate in the range of 0.5–1.5 equivalent monolayers (Eq-ML) per minute was estimated. The same evaporation conditions were used for all samples of one series.

The characterization of samples was carried out using XPS, confocal Raman microscopy, and AFM measurements. After each cobalt evaporation, in situ XPS core level spectra were performed with a CLAM4-MCD hemispherical electron analyzer (Thermo Fisher Scientific, Waltham, MA, USA), using a dual non-monochromatized Mg/Al Anode X-ray source, where alternative Mg Kα and Al Kα radiations were used to avoid overlapping of the main photoemission peaks and Auger lines. The pass energy was set at 20 eV, giving a spectral resolution of 0.9 eV for the Mg anode and 1 eV for the Al. The energy scale of XPS spectra was calibrated by adjusting the sp^2^ contribution of the graphene of the C 1s peak to 284.2 eV [34]. CasaXPS software was used to fit the XPS spectra, using Gaussian-Lorentzian product line-shape profiles and subtracting a Shirley-type background. The inelastic mean free path (IMFP) was estimated using the Tanuma, Powell, and Penn formula IMFP-TPP2M [35].

Raman spectra were measured with a confocal Raman microscope (ALPHA 300RA, WITec, Ulm, Germany) with laser excitation at 532 nm, a 100X objective lens (NA = 0.95), and an incident laser power of 2 mW. Samples were analyzed by mapping regions on the plane (XY scans, 20 × 20 μm^2^). Average Raman spectra were obtained from the in-plane scans, where a matrix containing 900 (30 × 30) sets of Raman spectra was recorded with an integration time of 1 s per spectrum in the spectral range 0–3600 cm^−1^. WITec Control Plus software was used to obtain spectra and intensity maps, whereas Raman bands were fitted by using CasaXPS software. Raman bands of each spectrum were fitted using Lorentzian functions.

The AFM images were taken with an AFM microscope from Nanotech Electrónica, Madrid, Spain, operating in dynamic mode (frequency modulation noncontact, FM-ncAFM), with frequencies around 300 kHz, and by using tips from NextTip, Madrid, Spain, with frequencies around 75 kHz. The images were processed and analyzed with the WSxM software [36].

## 3. Results

### 3.1. In Situ XPS Characterization of As-Grown CoO on Graphene

**Figure 1:**
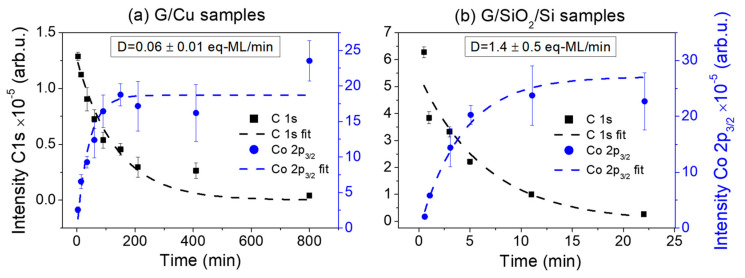
Intensity evolution of the C 1s and Co 2p_3/2_ regions for CoO deposition on (**a**) G/Cu and (**b**) G/SiO_2_/Si substrates.

Quantitative XPS analysis can be used to estimate the CoO thickness and, thus, the deposition rate by assuming a constant, layer-by-layer growth in the form of equivalent monolayers (eq-ML). Figure 1a,b show the peak area of C 1s and Co 2p_3/2_ peaks after background removal as a function of CoO deposition time for the CoO/G/Cu and CoO/G/SiO_2_/Si samples, respectively. A decrease/increase in C 1s and Co 2p_3/2_ signals, respectively, can be observed as the oxide is deposited, which is fitted using an exponential growth model of the form (1-exp(-d/λ)) for the overlayer peak and exp(-d/λ) for the substrate peak [37]. Assuming a constant evaporation rate for each set of samples, this model allows us to estimate the growth rate of the CoO deposits in terms of eq-ML/min [38] by using an estimated thickness of CoO monolayer of 2.1 Å, corresponding to the Co-O distance in the rock salt structure [39]. Therefore, deposition rates obtained for the G/Cu and G/SiO_2_/Si substrates using the C 1s signal are D_CoO/G/Cu_ = 0.06 ± 0.01 eq-ML/min and D_CoO/G/SiO2/Si_ = 1.4 ± 0.5 eq-ML/min, respectively, from which the coverage of CoO in ML can be obtained for each growth step.

**Figure 2:**
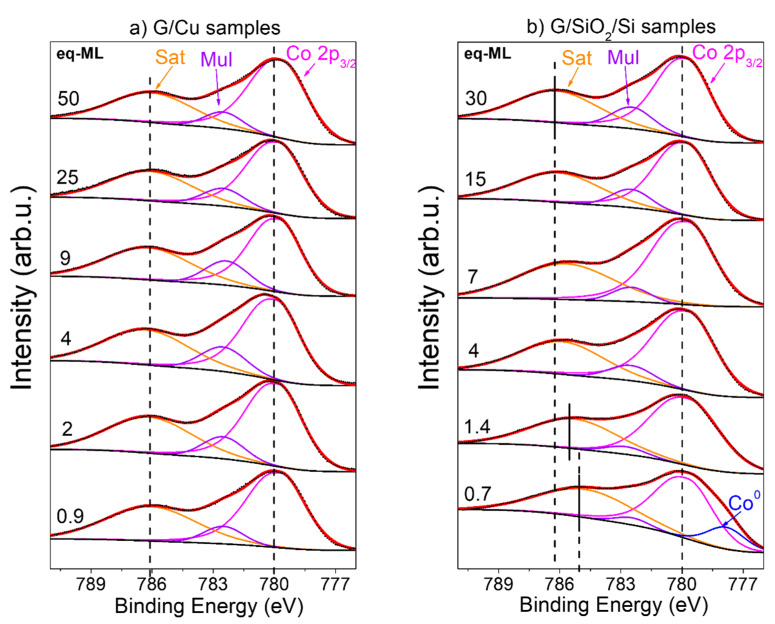
Co 2p_3/2_ XPS spectra of (**a**) G/Cu samples and (**b**) G/SiO_2_/Si samples (each sample coverage in eq-ML is shown at left). Experimental data and fitted spectra are presented with black dotted and continuous red lines, respectively. The black lines represent the Shirley-type background. The Orange, magenta, purple, and blue lines correspond to the individual fitting of the satellite (“Sat”), the multiplet (“Mul”), the main peak of CoO (“Co 2p_3/2_”) and the metallic contribution of Co (“Co”), respectively. The main line and satellite charge transfer positions are indicated by vertical dashed and continuous lines.

Figure 2a,b show the normalized spectra of Co 2p_3/2_ for both sets of samples, which were fitted with three components [28]: (i) the main peak at around 780 eV; (ii) the multiplet contribution at around 782.5 eV; and (iii) the charge transfer satellite with oxygen at around 786 eV, except for the first coverage on the G/SiO_2_/Si set of samples in which an additional peak corresponding to metallic Co (Co^0^) is identified at 788.6 eV. This latter peak is not present in the case of the G/Cu samples (Figure 2), and its presence has also been observed during the growth of Co oxides on SiO_2_ substrates [29].

The transfer satellite is a clear indication that the oxide formed is CoO, as expected for the growth condition in terms of temperature and partial oxygen pressure used in other works [27,28,29]. Furthermore, the main difference between both sets of samples is the progressive shift of the transfer satellite toward higher binding energies with increasing CoO coverage in G/SiO_2_/Si samples. In stark contrast, there is no shift in the satellite peak position in the G/Cu samples.

**Figure 3:**
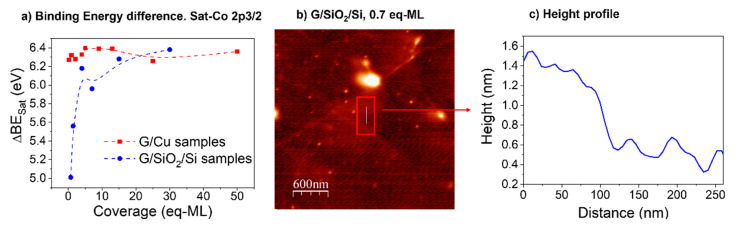
(**a**) Satellite-Co 2p_3/2_ binding energy separation (∆E_Sat_) for G/Cu and G/SiO_2_/Si samples (dashed lines guide the experimental points). (**b**) AFM topographic image (3 × 3 µm^2^) of G/SiO_2_/Si 0.7 CoO eq-ML sample, and (**c**) height profile on the line marked in Figure 3b.

The energy separation between the main line (Co 2p_3/2_) and satellite charge transfer (∆BE_Sat_) as a function of the CoO coverage for both series of samples is plotted in Figure 3a. It can be observed that the value corresponding to G/Cu samples (red symbols) remains almost fixed at 6.3 eV, while the value corresponding to G/SiO_2_/Si samples (blue symbols) grows from 5 eV to reach the same value as G/Cu samples, i.e., 6.3 eV.

From theoretical calculations [27,28], it is known that the increase in ∆BE_Sat_ relates to covalence gaining in the CoO. The low initial covalence seen for early stages of CoO growth (low values of ∆BE_Sat_) was previously associated with the formation of a CoO wetting layer in the CoO/HOPG system, whose low-dimensional character and strong interaction with HOPG [30] induces a break in crystalline symmetry. In the present case, the presence of the wetting layer in G/SiO_2_/Si samples is confirmed by AFM. Figure 3b shows the topographic image of 0.7 eq-ML of CoO on G/SiO_2_/Si, in which areas without well-defined borders but with different brightness can be distinguished, indicating different heights. A height profile extracted from the line marked in Figure 3b is shown in Figure 3c, where a height difference of about 8 Å is observed. This value corresponds to two-unit cells of the CoO rock salt structure, i.e., 4 eq-ML as previously defined (a = 4.261 Å for bulk CoO) [39]. The roughness of the SiO_2_ substrate does not allow the identification of a single wetting layer. However, the presence of these atomically thick plateaus with irregular borders is considered proof of the wetting layer formation, based on previous works [30]. It is worth mentioning that these plateaus are absent in coupled graphene (G/Cu) samples, which combined with the absence of a shift of the satellite-Co 2p_3/2_ point to an island growth model on G/Cu samples [28]. Moreover, the growth of CoO on bare SiO_2_ shows an initial decrease in ∆BE_Sat_ until its bulk value, confirming the key role of graphene monolayer [28].

The covalence is gained and stabilized after several CoO monolayers, as can be seen in Figure 3a. Thus, at high coverages (from 15 CoO eq-ML), CoO recovers its natural crystalline symmetry by growing CoO islands on top of the wetting layer (also confirmed later by AFM images). It is important to highlight that, for decoupled graphene, the growth of the wetting layer by self-assembly of CoO atomic clusters confirms the strong diffusion of these clusters found on HOPG substrates [27], replicated now in just one graphene layer. On the contrary, for coupled graphene, ∆BE_Sat_ indicates an island-model growth from the early stages of CoO coverage, as no significant changes in binding energy separation are noticeable (Figure 3a). This type of growth was confirmed by AFM analysis.

**Figure 4:**
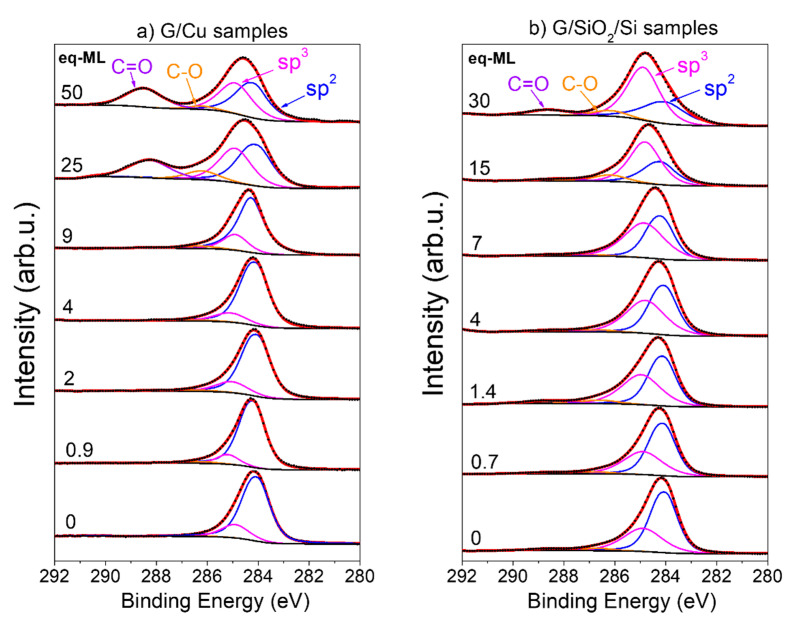
C 1s XPS spectra of (**a**) G/Cu and (**b**) G/SiO_2_/Si samples. Each sample coverage in eq-ML is shown on the left. Experimental data and fitted spectra are depicted with black dotted and red continuous lines, respectively. The black lines represent the Shirley-type background. Orange, purple, magenta, and blue lines correspond to the individual fitting of the C-O, C=O, sp^3^, and sp^2^ hybridization, respectively.

The evolution of the C 1s spectra for G/Cu and G/SiO_2_/Si sets is shown in Figure 4a,b. XPS spectra have been fitted with four well-known components [30,40]: (i) the sp^2^ hybridization (284.2 eV), the main carbon hybridization in graphene; (ii) the sp^3^ hybridization (285 eV) that is associated with crystalline disorder and defects in graphene layer (broken carbon hexagons); and iii) the C-O and C=O (286.3 eV and 288.5 eV, respectively). In more detail, the sp^2^ component can be fitted either by symmetric or asymmetric peak line shapes, where the second type modelizes neutralization by conduction electrons of the holes created during photoionization. As shown in our previous work [41], given the energy resolution of our equipment, very similar results were obtained following both methods, although a possible slight overestimation of graphene defects with sp^3^ hybridization can be expected. Therefore, we will use symmetric functions for the sake of simplicity.

**Figure 5:**
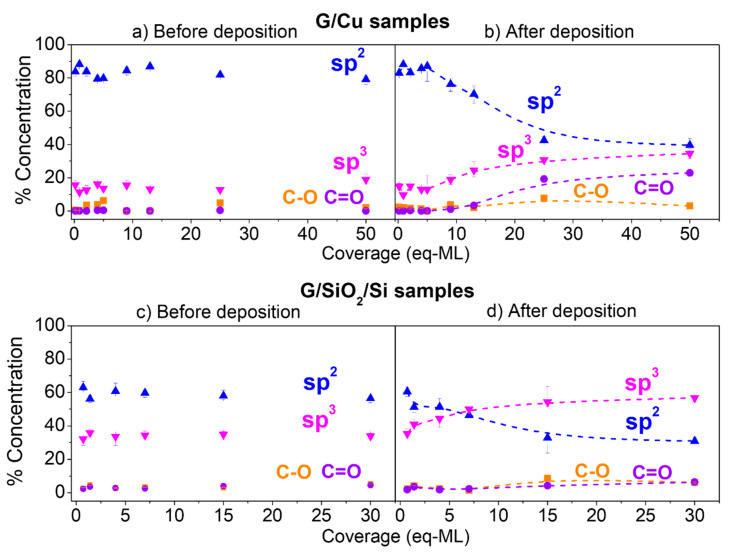
Evolution of each component (peak area over total) versus CoO coverage obtained from fitting the C 1s spectra for (**a**,**b**) G/Cu samples and (**c**,**d**) G/SiO_2_/Si samples. (**a**,**c**) contains concentration data before CoO growth, while (**b**,**d**) show component concentration after CoO deposition (dashed lines guide the experimental points).

To facilitate the study of the evolution of the different components with increasing coverage, the concentration of each one was determined by the component peak area over the total one. In addition, in order to analyze the effect of CoO growth on the chemical state of graphene and to discard different graphene initial states before CoO growth, the weight of the different components (in %) was estimated in the as-received G/Cu and G/SiO_2_/Si substrates (Figure 5a,c), and after CoO deposition in the G/Cu and G/SiO_2_/Si substrates (Figure 5b,d). In the case of the G/Cu substrates before deposition (Figure 5a), the graphene’s state is represented without any annealing treatment.

The relative concentrations before CoO growth (Figure 5a,c) indicate low oxygen contamination for both series of samples, as well as better structural quality for coupled graphene samples (G/Cu), i.e., less relative intensity of sp^3^ components, as expected for pristine graphene directly grown on Cu. The sp^2^ hybridization component shows a value of ~80% for all the G/Cu substrates used, whereas for decoupled graphene (G/SiO_2_/Si substrates), this component barely surpasses the 60%, likely related to the transfer process to the SiO_2_ substrate [42]. Since, for each set of samples, G/Cu and G/SiO_2_/Si, all substrates used present similar levels of sp^2^, sp^3^, C-O, and C=O percentage concentrations before the deposition, any change in graphene after CoO deposition can be straightforwardly related to the metal oxide/graphene interaction.

After CoO deposition, the different components suffer major changes. In the case of coupled graphene (G/Cu), the component related to graphene sp^2^ hybridization drops after the deposition of 5 eq-ML of CoO, whereas the component related to sp^3^ starts growing at the same point. Ultimately, both hybridizations reach a saturation state with a concentration of ~40%. For the decoupled graphene samples (G/SiO_2_/Si), similar behavior is found for both hybridizations, but, in this case, the effect of CoO deposition is almost instantaneous, observing the drop (increment) of sp^2^ (sp^3^) hybridizations from the very early stages of growth. In addition, due to the lower crystalline quality found in the base state of decoupled graphene, the concentration of sp^3^ hybridization rapidly surpasses the sp^2^ hybridization component, ending up with much more defective graphene for G/SiO_2_/Si samples as compared with the G/Cu samples. A final remark should be made on C-O and C=O concentrations: there is no significant oxidation for low and medium coverages in both sample series and only it is observed in the highly covered samples. For this last case, the higher values of C=O concentration, which are the dominant oxidation state, can be related to the oxidation of graphene promoted by CoO growth, as previously observed for HOPG [30].

### 3.2. Defects Characterization of Graphene by Confocal Raman Microscopy

**Figure 6:**
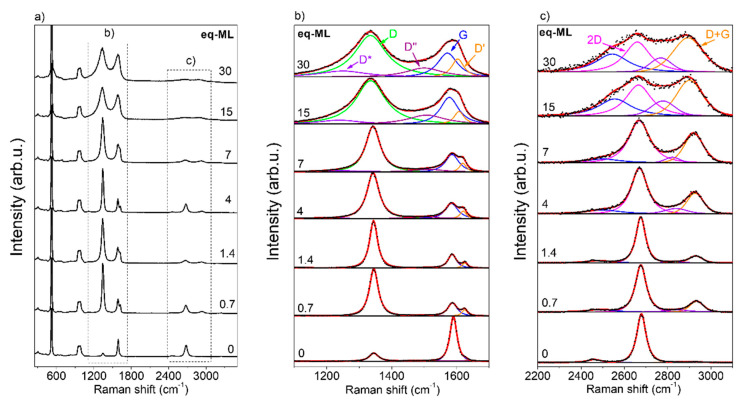
(**a**) Average Raman spectra obtained of in-plane mapping of G/SiO_2_/Si samples with different CoO coverages. (**b**,**c**) shows the graphene bands of (**a**), deconvoluted by Lorentzian curves. Experimental data and fitted spectra are presented by black dotted and red continuous lines, respectively. In (**b**), green, orange, maroon, purple, and blue lines correspond to the individual fitting of D, D′, D″, D*, and G, respectively. In (**c**), magenta and orange lines correspond to the individual fitting of 2D and D + G, respectively.

Raman spectroscopy has been extensively used to identify disorders and defects on graphene and graphene oxide (GO) [43,44,45,46,47]. It is a complementary technique to XPS since the latter only allows identifying defects with sp^3^ hybridization and oxidation. Figure 6a shows the Raman spectra of the different CoO coverages deposited on G/SiO_2_/Si samples in the spectral range of 250 cm^−1^ to 3500 cm^−1^, showing Raman active bands due to SiO_2_, silicon substrate, and graphene. The Raman scattering of Co-O vibrations is not distinguished in our spectra due to the low scattering of the rock salt CoO phase and the overlapping with the intense signal from the silicon substrates [48]**.** The diamond structure of silicon allows the presence of one Raman active phonon mode located at the center of the Brillouin zone (BZ), corresponding to a wavenumber of 520 cm^−1^ (LTO) and a broad peak at 935–985 cm^−1^, due to the scattering of several transverse optical phonons (2TO) and their overtone state. The asymmetric band around 550 cm^−1^ corresponds to the longitudinal optical (LO) [49]. The rest of the vibrational Raman modes correspond to the graphene structure [43,46,47]. The D band at 1345 cm^−1^ and the D′ band at 1628 cm^−1^ are Raman-forbidden bands activated when defects allow momentum conservation in the Raman scattering process. The D band, also called the D disorder-induced band, arises from the defects and disorders in the carbon lattice and the double-resonant processes near the K point of the BZ boundary [46,47]. The G band at 1580 cm^−1^ originates from the E_2g_ optical phonon mode, and it is the only band coming from a normal first-order Raman scattering process in graphene. The D″ band at 1500–1550 cm^−1^, is related to the amorphous phase, and its intensity is inversely related to the crystallinity, while the D* band (1247 cm^−1^) is related to the existence of sp^3^ bonds [46,47]. In GO, these two bands, D″ and D*, are related to the oxygen content [44]. The 2D band, also called the G′ band, is approximately twice the D band frequency and originates from a second-order process involving two phonons near the K point, being allowed in graphene without any disorder or defects [46]. It has been used to distinguish a single graphene layer from multilayers of graphene [47].

It should be mentioned that due to the high fluorescence observed in G/Cu samples, even though it is possible to distinguish the D, G, and 2D bands [50], it was not possible to quantify their evolution with CoO coverage, so we focused our study only on the G/SiO_2_/Si samples.

Figure 6b,c show the evolution of Raman spectra of graphene, as the CoO coverage increases, showing the deconvolution of the first-order Raman modes: D*, D, D″, G, and D′ bands (Figure 6b) and the second-order Raman modes, mainly 2D and D + G (Figure 6c). Strong changes in the intensity, position, and full width at half maximum (FWHM) of the Raman bands are identified with increasing the CoO coverage. On the one hand, for the lowest value of the CoO coverage, a significant increase in the D band along with the appearance of the D′ mode is identified with respect to the pristine graphene. On the other hand, from 15 eq-ML of CoO, the Raman spectrum varies significantly: a clear width of the D and G bands is observed in addition to the identification of the D* and D′ Raman modes (see Figure 6b).

The ratio between intensities and areas of these Raman bands was used intensively to study the disorder, the number of defects, and their nature (vacancies, grain boundaries, and sp^3^ bonds) in graphene [43,51], as well as the oxidation of graphene and the reduction of GO [44,45]. It is well established that the D/G ratio provides information related to the density of graphene defects [43,47,51], while the D/D′ ratio gives information on their nature [43,51]. In pristine graphene, the I(2D)/I(G) ratio is an indication of the number of graphene layers, being also used to identify nanographene or polycrystalline graphene [46,47]. In the case of the oxidation of graphene or reduction of GO, the I(D*)/I(G) is related to the C/O ratio [44].

**Figure 7:**
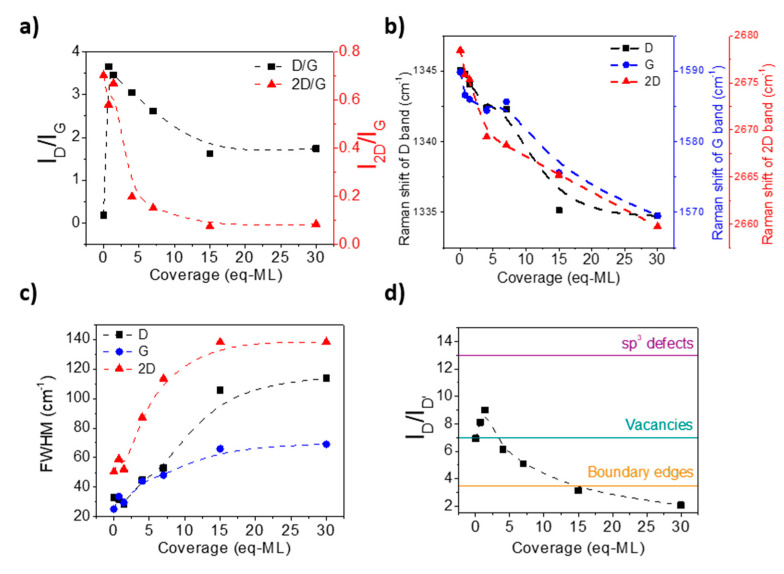
(**a**) I(D)/I(G) and I(2D)/I(G) intensity ratios of Raman spectra as a function of CoO coverage in eq-ML for G/SiO_2_/Si samples. (**b**) Raman shift in D, G, and 2D bands as a function of CoO coverage in eq-ML for G/SiO_2_/Si samples. (**c**) FWHM of D, G, and 2D bands as a function of CoO overage in eq-ML for G/SiO_2_/Si samples. (**d**) I(D)/I(D′) intensity ratio of Raman spectra as a function of CoO coverage in eq-ML for G/SiO_2_/Si samples. Horizontal continuous lines indicate the I(D)/I(D′) ratio associated with sp^3^ defects (≃13), vacancy-like defects (≃7), and boundary-like defects (≃3.5). Dashed lines guide the experimental points.

Figure 7a shows the evolution of the I(D)/I(G) and I(2D)/I(G) ratios as a function of the CoO equivalent monolayers deposited on G/SiO_2_/Si samples. A strong increase in the I(D)/I(G) ratio for the first two CoO coverages is observed, which decreases as the CoO eq-ML increases. This is an unexpected behavior if we compare it with the increase in sp^3^-related defects observed by XPS (Figure 5d). A similar trend of the I(D)/I(G) signal as the number of defects increases has been previously reported by Z. Luo et al. [52] inducing defects on graphene by hydrogenation, and by Lucchese et al. [53] developing defects by Ar^+^ bombardment and explaining this behavior in terms of a local active model of the D band. This model assumes that around a defect there are two regions: the first, closer to the defect that corresponds to a structurally disordered region in which the break of the hexagonal crystalline structure occurs; and a second region surrounding the disorder structural region, called activated region, in which the lattice structure is preserved, but due to the proximity of a structural defect an enhancement of the D band takes place. As the defect density increases, activated regions overlap, and the intensity of the D band reaches a maximum. This occurs when the mean distance between defects, *d*, is equal to r_A_ + r_s_ (r_s_ is the radius of the disorder structural region and r_A_ is the size of the activated region). If the defect density keeps increasing, the defects coalesce into a bigger defect and the graphene starts to be dominated by structural disorder areas with hexagonal lattice shrink that induces a decrease in the intensity of the D band [52,53]. Therefore, the saturation observed in I(D)/I(G) for higher CoO coverages is an indication of highly defective graphene.

The I(2D)/I(G) signal is also represented in Figure 7a, which stays approximately constant for small CoO coverages and shows an abrupt decrease for 4 eq-ML of CoO, reaching saturation around 15 eq-ML of CoO. It has been found that the intensity of I(2D)/I(G) is maximum at zero chemical doping of graphene, i.e., an absence of charges at the surface and interface [54]. On defective graphene, the electron-defect collision, in addition to electron–electron and electron–phonon collisions, contributes to the total scattering rate causing a decrease in the I(2D)/I(G) signal [52]. However, as Eckmann et al. have observed, the dependence on defects of the defect-activated bands, D and D′, and the two-phonon Raman band 2D is different [43]. A constant intensity of the 2D band is observed when the number of defects is low enough. However, it decreases when the effect of the reduction in the electron lifetime due to defects, related to the decrease in the inter-defect distance, is considerably important. This behavior seems to be very similar to our observations. The stabilization of the I(2D)/I(G) intensity ratio for the highest CoO coverages seems to be related to the increase in other defect-related bands as first-order D* and D″ bands as well as the presence of the C=O and C-O bonds, as is shown on Figure 5d.

In addition, as defects are generated by CoO deposition, more strain accumulates in the graphene lattice, and larger redshifts are seen for higher coverages. Figure 7b shows the Raman shift for D, G, and 2D bands obtained from peak positions corresponding to the sample average spectra: a redshift of about 20 cm^−1^ for G and 2D bands and ~10 cm^−1^ for the D band from the pristine G/SiO_2_/Si sample to the highest coverage. This redshift has been reported before by X. Zheng et al. [55], where electrostatic attractive forces between graphene and gold nanoparticles create tensile strains in the graphene layer, breaking the symmetry of the graphene lattice by stretching carbon bonds and producing Raman bands redshifting. The increase in FWHM of the D, G, and 2D bands with the CoO coverage (see Figure 7c) also indicates the increase in the defect number due to CoO deposition on uncoupled graphene. This increase is related to the minor phonon lifetime due to defects scattering [52]. In addition, as commented above, the appearance of the D″ mode is noted from 15 eq-ML of CoO, indicating the amorphous character of the graphene with a black carbon-like structure [56].

Finally, to identify the type of defects induced by CoO deposition, Figure 7d shows the I(D)/I(D′) intensity ratio as a function of CoO coverage. It was established that I(D)/I(D′) is the maximum (≃13) for defects associated with sp^3^ hybridization, decreasing for vacancy-like defects (≃7), and reaching a minimum for boundary-like defects in graphite (≃3.5) [43,51]. In our case, the obtained I(D)/I(D′) values are lower than expected for sp^3^ defects, indicating that apart from sp^3^ hybridization defects, other kinds of defects, such as vacancies or domain boundaries, are present in the samples, which predominate at higher CoO coverages.

**Figure 8:**
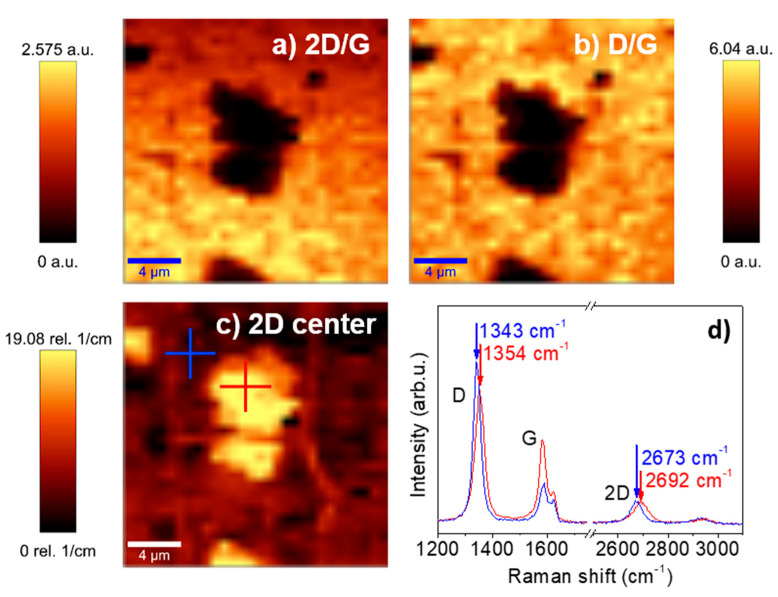
(**a**) 2D/G intensity ratio, (**b**) D/G intensity ratio, and (**c**) Raman shift in the 2D band for the 0.7 eq-ML G/SiO_2_/Si sample Raman maps (size: 20 × 20 μm^2^). (**d**) Raman spectra extracted from marked red and blue crosses in Figure 8c.

In addition, from Raman mappings, inhomogeneous zones related to the graphene signal are identified. Figure 8a,b represent the Raman maps corresponding to the I(2D)/I(G) and I(D)/I(G) intensity ratios, where the darkest regions are associated with low values of intensity ratios. For the sake of clarity, red and blue crosses have been marked on the maps, and the corresponding Raman spectra are shown in Figure 8d. The blue cross corresponds with higher values of 2D/G and D/G ratios, due to the detriment of G band intensity, which is directly related to a lower crystalline quality of graphene, while the red cross is situated in a highly crystalline quality region since the G band reaches top values. These brighter regions correspond to defective graphene areas. The Raman shift position of the 2D band is shown in Figure 8c, with a redshift of 23 cm^−1^ compared to the 2D position found on the blue cross. A redshift is also observed on the D Raman band comparing both areas; however, the G band remains unshifted. Previous works have studied G and 2D bands redshifts as a consequence of uniaxial strain applied to graphene on flexible substrates [57], and local radial strain generated by nanometric particles [58], with the 2D band more sensitive. Two-dimensional redshift can also be a consequence of graphene n-type doping, but G blueshift should be expected in this case [59]. However, we do not identify any Raman shift for the G band of 0.7 eq-ML sample (similar behavior for all other samples). However, a significant high shift for the 2D band is observed, which is in agreement with the results found by Mohiuddin et al. [57] where redshifts up to 90 cm^−1^ for uniaxial strain graphene are reported. Thus, even if it is not possible to observe the CoO signal in the Raman spectra, the Raman mappings obtained for the 2D/G and D/G intensity ratios and for 2D Raman shift, shown in Figure 8, allow local defects on graphene induced by CoO deposition to be identified. Raman mappings reveal that strained graphene zones (darker ones in Figure 8c) correspond to defective graphene areas (brighter ones in Figure 8a,b), showing a correspondence between defects and strain.

### 3.3. AFM Characterization of As-Grown CoO on Graphene

**Figure 9:**
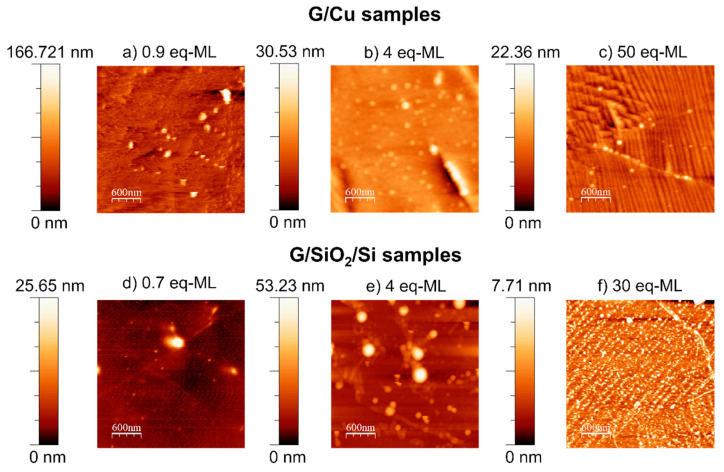
AFM topographic images obtained by noncontact dynamic mode for G/Cu samples: (**a**) 0.9 CoO eq-ML, (**b**) 4 CoO eq-ML, and (**c**) 50 CoO eq-ML; and G/SiO_2_/Si samples: (**d**) 0.7 CoO eq-ML, (**e**) 4 CoO eq-ML, and (**f**) 30 CoO eq-ML. Size: 3 × 3 µm^2^. The scale height bar for each image has been included.

AFM topographic images for three different coverages of the G/Cu and G/SiO_2_/Si series are shown in Figure 9, and the corresponding RMS roughness is included in Table 1. For low coverages (see Figure 9a,d), CoO is presented as bright circular islands with different diameters and heights. For both samples, the diameter of the islands ranges between 40 and 250 nm, whereas heights show major differences, from 5 to 25 nm on decoupled graphene and from 30 to 170 nm in coupled graphene. As discussed before, the presence of a wetting layer, confirmed by the satellite-Co 2p_3/2_ binding energy separation evolution (shown in Figure 3a), and by AFM (Figure 3b) for G/SiO_2_/Si samples, indicates a Stranski-Krastanov growth mode on decoupled graphene. A similar result was found during the growth of CoO on graphite when using the same CoO deposition conditions [30]. On the contrary, AFM topographic measurements for the G/Cu samples confirm a 3D island growth mode for coupled graphene.

Medium coverages (see Figure 9b,e) show significant concentrations of CoO islands. In this case, islands with heights of 5–50 nm and diameters of 100–300 nm were found in both samples. In addition, the growth of lower structures that interconnect islands in the G/SiO_2_/Si sample is observed. Intriguingly, close to coalescence into a continuous layer, the CoO nanoparticles seem to form strip-like structures, with dimensions ranging between 70 and 150 nm.

The RMS roughness, included in Table 1, for G/Cu samples, decreases from the 0.9 eq-ML sample to the highest coverage sample. However, in the case of the G/SiO_2_/Si samples, the roughness reaches a maximum at 4 eq-ML coverage. The overall roughness for the lowest coverages is lower in the case of the G/SiO_2_/Si samples, i.e., 1.48 nm, with respect to the G/Cu ones, i.e., 7.90 nm, and this could be related to the formation of the wetting layer at the first coverage. With the formation and accumulation of the CoO islands on top of the wetting layer, roughness increases as can be seen for the 4 eq-ML sample. For the final coverages, similar values of RMS roughness are seen for coupled and decoupled graphene samples, and in both, similar-size strip-like structures are observed.

## 4. Discussion

Our results clearly indicate a Stranski-Krastanov growth mode for CoO grown on decoupled graphene and a Volmer–Weber mode on coupled graphene. The Stranski-Krastanov growth mechanism is frequently found in systems where adatom–surface interaction is more intense than adatom–adatom interaction. The electronic coupling between graphene and its support weakens the interaction between graphene and CoO, preventing larger diffusion rates of the CoO clusters and forming nanometric islands in G/Cu samples instead of a wetting layer for the first coverages.

The analysis of C-O and C=O concentrations indicates that there is no oxidation for low and medium coverages of CoO on either, coupled or decoupled graphene, but there are small concentrations for the highest coverages samples. Comparing similar coverages, the 25 eq-ML G/Cu sample exhibits higher C=O concentration (~20%) than the 30 eq-ML G/SiO_2_/Si sample (~10%), not observing any oxidation signals on copper. In the case of deposition of ZnO on G/Cu graphene [40], it has been found that the oxidation of graphene is due to the cathodic nature of copper that promotes the oxidation of graphene in which the intercalated species between Cu and graphene due to air exposure as oxygen and water molecules play an important role. However, this is not the case in our G/Cu samples since the graphene was cleaned in UHV conditions, and the deposited CoO probably induced the fast oxidation of graphene, avoiding the galvanic corrosion of copper. In both sets of samples, the emergence of the C=O contribution in the C 1s spectra at similar coverages demonstrates that CoO promotes the oxidation of graphene.

The analysis of the Raman spectra for CoO deposited on G/SiO_2_/Si samples shows a dependence of the graphene bands with the CoO coverage. A more defective graphene structure is found as the CoO coverage increases. From the Raman mappings, the identification of regions’ more defective regions is identified, revealing a correlation between defects and strain in the graphene lattice. Although a more detailed study must be conducted to analyze how the developed defects induce strain in the graphene lattice, it has been confirmed that the concentration of defects, including the ones related to sp^3^ hybridization, generates strain in the samples confirmed by the larger redshifts in the D, G, and 2D bands as the CoO coverage increases.

Finally, the unexpected formations of strip-like structures for the highest CoO coverages observed by AFM in both kinds of substrates (Figure 9c,f for G/Cu and G/SiO_2_/Si substrates, respectively) have not been reported before and discard the coupling state of graphene as responsible. More experiments should be performed in order to understand the formation mechanism of these nanostructures.

## 5. Conclusions

In this work, the CoO interaction with coupled and decoupled graphene was studied as a function of the deposited CoO coverage. The initial interaction of graphene with the substrate plays an important role in the interaction of CoO with graphene. In fact, regarding CoO growth morphology, two different growth modes are reported depending on the graphene/substrate initial electronic coupling state: the Volmer-Weber model on coupled graphene and the Stranski-Krastanov growth mechanism on decoupled graphene, identifying the CoO wetting layer formed. The electronic coupling between graphene and its support seems to relax the interaction between graphene and CoO, preventing larger diffusion rates of the CoO clusters and forming nanometric islands in G/Cu samples instead of a wetting layer for the first coverages. Moreover, XPS results show an increment of sp^3^-like defects in graphene, while Raman analysis indicates that other kinds of defects with sp^2^ hybridization are also induced by CoO deposition, such as vacancies or domain boundaries. Moreover, the gradual oxidation of graphene induced by CoO becomes critical for relatively high CoO coverages. Independently of the substrate, these results indicate a C-C bond weakening similar to that induced by CoO on HOPG.

## Figures and Tables

**Table 1:** RMS roughness for G/Cu samples and G/SiO_2_/Si samples included in Figure 9.

	Coverage (eq-ML)	RMS Roughness (nm)
G/Cu	0.9 4 50	7.9 2.82 1.86
G/SiO_2_/Si	0.7 4 30	1.48 6.07 1.22

## Data Availability

Data will be made available upon reasonable request.
